# Laparoscopic Liver Resection Should Be a Standard Procedure for Hepatocellular Carcinoma with Low or Intermediate Difficulty

**DOI:** 10.3390/jpm11040266

**Published:** 2021-04-02

**Authors:** Ruoh-Yun Gau, Ming-Chin Yu, Hsin-I Tsai, Cheng-Han Lee, Tony Kuo, Kuan-Chieh Lee, Wei-Chen Lee, Kun-Ming Chan, Chien-Chih Chiu, Chao-Wei Lee

**Affiliations:** 1 Division of General Surgery, Department of Surgery, Linkou Chang Gung Memorial Hospital, Guishan, Taoyuan 333, Taiwan; poclee24@gmail.com (R.-Y.G.); weichen@adm.cgmh.org.tw (W.-C.L.); chankunming@adm.cgmh.org.tw (K.-M.C.); 2 College of Medicine, Chang Gung University, Guishan, Taoyuan 333, Taiwan; mingchin2000@gmail.com (M.-C.Y.); tsaic@hotmail.com (H.-I.T.); b9102011@cloud.cgmh.org.tw (C.-H.L.); B9302028@cgmh.org.tw (T.K.); guanjie0826@gmail.com (K.-C.L.); 3 Graduate Institute of Clinical Medical Sciences, Chang Gung University, Guishan, Taoyuan 333, Taiwan; 4 Department of Surgery, New Taipei Municipal Tu-Cheng Hospital (Built and Operated by Chang Gung Medical Foundation), Tu-Cheng, New Taipei City 236, Taiwan; 5 Department of Anesthesiology, Linkou Chang Gung Memorial Hospital, Guishan, Taoyuan 333, Taiwan; 6 Department of Gastroenterology and Hepatology, Linkou Chang Gung Memorial Hospital, Guishan, Taoyuan 333, Taiwan; 7 Department of Nursing, Linkou Chang Gung Memorial Hospital, Taoyuan 333, Taiwan; liver8.tw@gmail.com

**Keywords:** laparoscopic, liver resection, hepatectomy, hepatocellular carcinoma, hepatoma, difficulty score, IWATE criteria

## Abstract

Background: To investigate the feasibility of laparoscopic liver resection (LLR) for hepatocellular carcinoma (HCC), we compared the outcome between LLR and conventional open liver resection (OLR) in patient groups with different IWATE criteria difficulty scores (DS). Methods: We retrospectively reviewed 607 primary HCC patients (LLR: 81, OLR: 526) who underwent liver resection in Linkou Chang Gung Memorial hospital from 2012 to 2019. By using 1:1 propensity score-matched (PSM) analysis, their baseline characteristics and the DS stratified by the IWATE criteria were matched between the LLR and OLR. Their perioperative and oncologic outcomes were compared. Results: After 1:1 PSM, 146 patients (73 in LLR, 73 in OLR) were analyzed. Among them, 13, 41, 13 and 6 patients were classified as low, intermediate, advanced and expert DS group, respectively. Compared to OLR, the LLR had shorter hospital stay (9.4 vs. 11.5 days, *p* = 0.071), less occurrence of surgical complications (16.4% vs. 30.1%, *p* = 0.049), lower rate of hepatic inflow control (42.5% vs. 65.8%, *p* = 0.005), and longer time of inflow control (70 vs. 51 min, *p* = 0.022). The disease-free survival (DFS) and overall survivals were comparable between the two groups. While stratified by the DS groups, the LLR tended to have lower complication rate and shorter hospital stay than OLR. The DFS of LLR in the intermediate DS group was superior to that of the OLR (*p* = 0.020). In the advanced and expert DS groups, there were no significant differences regarding outcomes between the two groups. Conclusion: We have demonstrated that with sufficient experience and technique, LLR for HCC is feasible and the perioperative outcome is favorable. Based on the current study, we suggest LLR should be a standard procedure for HCC with low or intermediate difficulty. It can provide satisfactory postoperative recovery and comparable oncological outcomes. Further larger scale prospective studies are warranted to validate our findings.

## 1. Introduction

As a promising minimally invasive technique, laparoscopic liver resection (LLR) has been widely adopted in recent decades [1]. Compared to the conventional open liver resection (OLR), the laparoscopic approach provides several advantages such as reduced blood loss, shorter postoperative length of stay (LOS) and lower complication rates [2,3,4,5,6,7]. The indications for LLR have thus gradually extended from benign lesions to malignant ones, including hepatocellular carcinoma (HCC). HCC is the most common primary malignancy of the liver, and it ranks among the top three leading causes of cancer-related death worldwide [8]. Owing to the application of semiannual surveillance program, the increased proportion of tumors diagnosed at an earlier stage, and advancing surgical techniques as well as perioperative care, the overall survival of HCC patients after liver resection is improving in the recent decades [9]. As a result, liver resection remains one of the most effective curative treatments for HCC.

As for LLR per se, despite comparable short-term outcomes, it is still under investigation whether the laparoscopic approach is justified for HCC in terms of the long-term oncologic results, such as resection margin, recurrence rates and survival. Several studies reported promising perioperative results without compromise in the oncologic outcomes for LLR [3,7,10,11,12,13]. However, the heterogeneity of the patient population, disease status, extent of liver resection, and procedural type complicated the assessment for LLR. Although the dichotomy of minor/major liver resection was the most commonly used terminology to stratify the complexity of LLR, it has been manifested that many other factors, such as tumor location and underlying liver cirrhosis, would influence the outcome after liver resection [14]. It was therefore concluded by the second international consensus conference on LLR (ICCLLR) in 2014 that a preoperative difficulty scoring system comprising the extent of liver resection, tumor location, tumor size, proximity to major vessels, and the severity of fibrosis is mandatory to select and protect the patients undergoing LLR [15,16,17]. The IWATE criteria, a modified four-level difficulty scoring system based on previous three-level difficulty scoring system constructed in 2014, was proposed by Professor Go Wakabayashi thereafter [18]. This novel difficulty scoring system has been validated by several encouraging studies with promising predictive values for LLR [19,20,21]. Despite the remarkable results, a head-to-head comparison study is still lacking to address the outcomes of LLR and OLR for HCC cases with different difficulties. The aim of this study, by adopting this novel IWATE criteria, was to investigate the feasibility and effectiveness of LLR for HCC. By comparing the outcomes between these two surgical modalities, we would like to establish a statement regarding the position of LLR for HCC.

## 2. Methods

### 2.1. Patients

**Figure 1:**
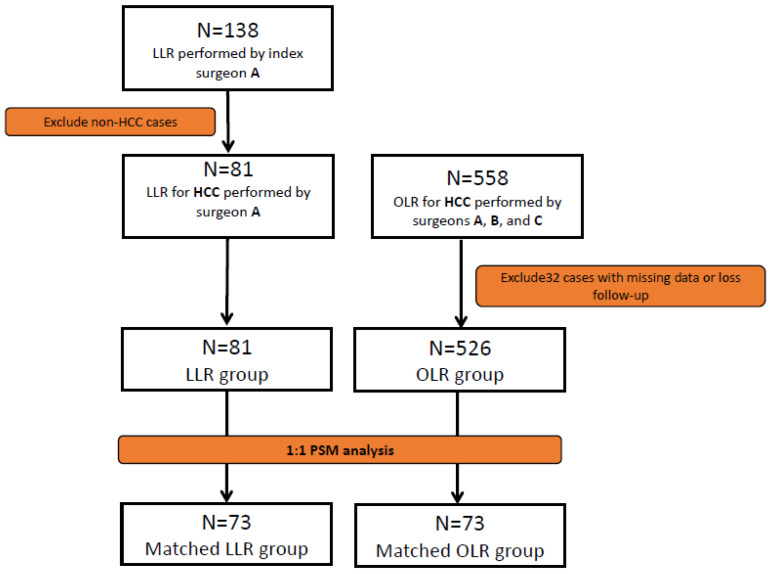
Flow diagram of the current study. Patients who underwent either laparoscopic liver resection (LLR) or open liver resection (OLR) for their HCC were reviewed. Propensity score-matched analysis (PSM) was conducted to compare the outcome between LLR and OLR.

Figure 1 is the flow diagram of the current study. Between 2012 and 2019, a total of 138 LLR were conducted for various indications by the same surgical team at Linkou Chang Gung Memorial Hospital (CGMH). The patients’ demographic data and surgical outcome were recorded. The fundamental operative setting and surgical procedure were described in our previous report [7]. To evaluate the feasibility and effectiveness of LLR for HCC, we further focused our analysis upon 81 of these 138 patients whose final pathology was confirmed to be HCC. To compare the outcomes, the HCC patients who underwent OLR with the same surgical team during the same period were enrolled. Five hundred and fifty-eight patients who received OLR for HCC were reviewed.

The criteria used to define resectable disease remained consistent over the entire study period: a lack of cancerous thrombi in the main trunk of the portal vein, no distant metastasis to other organs, a technically operable main tumor, an adequate liver functional reserve, and a sufficient future liver remnant. The liver functional reserve was assessed preoperatively by both Child–Pugh classification and indocyanine green (ICG) retention test at 15 min (ICG-15). In our institute, an ICG-15 ≤ 10% was the prerequisite for major hepatic resection [22]. Major hepatic resection was based on the Brisbane 2000 terminology and defined as the resection of three or more liver segments [14]. Cases with additional extrahepatic abdominal surgery (except cholecystectomy), vascular/biliary reconstruction, loss of follow-up, or inability to sum the IWATE score due to lack of index data were excluded from the subsequent analysis. A total of 608 HCC patients, comprising 81 in the LLR group and 527 in the OLR group, were included. To overcome the potential selection bias, a 1:1 propensity-score matched (PSM) analysis adjusted for various index variables including age, gender, diabetes mellitus, hypertension, liver cirrhosis, AJCC cancer stage, and the index parameters proposed by the IWATE criteria were adopted to minimize the differences between the two groups. After PSM, 73 matched pairs, consisting of 13, 44, 13, and 6 in low, intermediate, advanced, and expert difficulty groups, respectively, were generated. The prognosis in each difficulty group was also analyzed in an attempt to determine the safety and feasibility of LLR for HCC of different difficulty levels. The study was approved by the Institutional Review Boards (IRB) of Chang Gung Memorial Hospital (CGMH IRB No: 202000779B0).

### 2.2. Definition

Surgical duration was defined as the time period elapsing from the start of anesthesia induction to extubation. The severity of postoperative complications was graded according to the Clavien–Dindo classification, and grade III and IV complications were classified as major complications [23,24,25,26]. In-hospital mortality defined the occurrence of death during the same hospital stay. Recurrence was defined as the appearance of typical radiologic findings during regular postoperative imaging examinations. Disease-free survival (DFS) was the time interval from the date of surgery to the date of the first documented disease recurrence. Overall survival (OS) was the time period from the date of surgery to either the date of death or the date of the last follow-up.

The parameters of the IWATE criteria included tumor location, tumor size (<3 or ≥3 cm), extent of liver resection (partial resection, left lateral resection, segmentectomy, sectionectomy, or more), liver function (Child–Pugh score A/B), proximity to major vessels (distance of tumor to major hepatic veins, inferior vena cava, or main branches of Glisson’s tree less than 1 cm), and use of the hybrid approach or hand-assisted laparoscopic surgery (HALS). The difficulty was categorized into four levels according to the index score: scores 1–3, 4–6, 7–9, and 10–12 as low, intermediate, advanced, and expert difficulty score (DS) group, respectively [21].

### 2.3. Statistical Analysis

Continuous variables were analyzed by Student’s *t* test or Mann–Whitney U test, while categorical variables were assessed by Pearson’s χ^2^ test or Fisher’s exact test. Significant variables found by the univariate analysis were subjected to logistic regression analysis to identify independent risk factors. Kaplan–Meier analysis and the log-rank test were used to determine the OS and DFS. Statistical significance was defined as *p* value < 0.05. The statistical analysis was carried out using IBM SPSS Statistics 22 (IBM Corporation, Software Group, Somers, NY, USA).

## 3. Results

### 3.1. Demographic Characteristic of 138 Patients Who Underwent LLR

The patient demography was summarized in Table 1. The mean age was 57 years and more than 65% were male. Hepatocellular carcinoma was the most common diagnosis (*n* = 81, 58.7%), followed by benign hepatic tumor (*n* = 32, 15.2%) and metastatic liver tumor (*n* = 15, 10.9%). The mean tumor size was 3.78 cm and about 16% of tumors were larger than 5 cm. The intermediate DS group accounted for more than half of the cases, followed by the advanced and low DS groups (21% and 18.8%, respectively). The overall postoperative complication rate was 14.5%, and three cases (2.2%) developed major complications. There was no in-hospital mortality.

To identify risk factors associated with the development of postoperative complications, statistical analysis was conducted and the results are summarized in Table 2. Diabetes mellitus (DM), hypertension, liver cirrhosis, preoperative anemia (hemoglobin < 12 g/dL), and tumor located at segment 7/8 of liver were found to be related to the occurrence of postoperative complications. After multivariate logistic regression analysis, only anemia and tumor location were independently associated with the development of complications (*p* = 0.012 and 0.015, respectively). The extent of liver resection or the diagnosis of HCC, on the other hand, was not related to postoperative complications.

### 3.2. LLR and OLR for HCC before PSM

As shown in Table 3, 81 patients received LLR and 526 patients received OLR for their HCC. Most of the patient characteristics were comparable between the two groups, except that more patients in the OLR group had undergone previous abdominal surgery prior to the index operation (27.6 vs. 11.1%, *p* = 0.001). HBV infection remained the most common cause of HCC in both groups (around 60%), followed by HCV infection. About 45% of the patients in both groups had cirrhosis, and the majority of them were Child–Pugh grade A. The tumor features, however, were significantly different between the two groups. The tumors in the LLR group tended to be smaller (3.16 ± 1.44 vs. 5.13 ± 3.83, *p* < 0.001), less often located at segment 7 or 8 (17.3% vs. 50.8%, *p* < 0.001), and receiving less major resection (14.8% vs. 35.0%, *p* < 0.001). The tumors in the OLR group, on the other hand, had more vascular invasion (*p* = 0.008) and were far more advanced in terms of cancer stage (*p* = 0.005). The distribution of difficulty levels defined by the IWATE criteria was also different between the two groups; there were more patients in the OLR group allocated into either the advanced or expert levels (*p* < 0.001).

**Figure 2:**
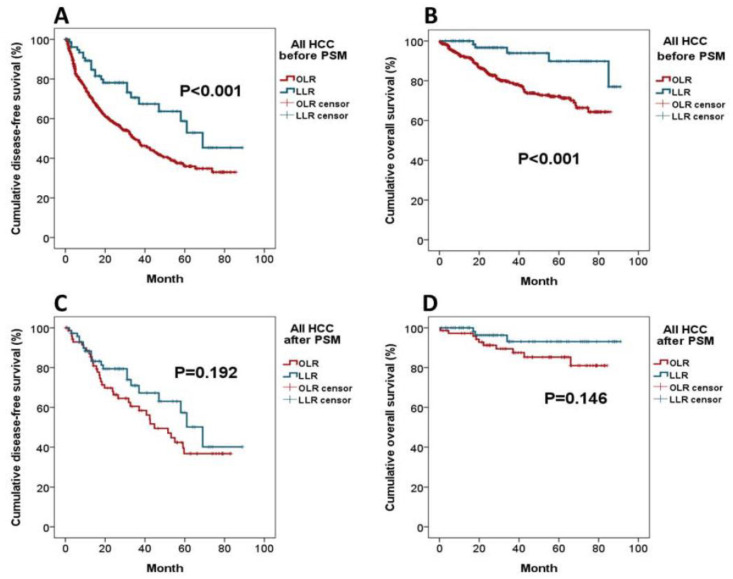
Kaplan–Meier survival analysis of LLR and OLR for HCC. (**A**) Disease-free survival (DFS) curves before PSM; (**B**) Overall survival (OS) curves before PSM; (**C)** DFS curves after PSM; and (**D**) OS curves after PSM.

Compared to the OLR, the LLR group had better perioperative outcome including lower postoperative complication rate (16.0% vs. 35.2%, *p* = 0.001), lower major complication rate (2.5% vs. 9.3%, *p* = 0.049), smaller amount of intraoperative blood loss (336.3 vs. 575.0 mL, *p* < 0.001), and shorter postoperative hospital stay (9.3 vs. 12.0 days, *p* < 0.001). Moreover, the LLR group enjoyed a significantly better DFS (median DFS 59 ± 4.7 vs. 43 ± 1.8 months, *p* < 0.001) and OS (median OS 84 ± 2.7 vs. 67 ± 1.5 months, *p* <0.001) than the OLR group (Figure 2A,B).

### 3.3. LLR and OLR for HCC after PSM

After PSM, there were 73 matched patients in both groups. The baseline clinical characteristics including comorbidities, previous history of abdominal surgery, preoperative liver reserve, resection extent, and tumor features were comparable between the two groups (Table 4). The patients in each group were allocated into either low (*n* = 13, 17.8%), intermediate (*n* = 41, 56.2%), advanced (*n* = 13, 17.8%) and expert (*n* = 6, 8.2%) DS groups according to the IWATE criteria. Except for more daughter nodules in the LLR group, the pathological features as well as the cancer staging were equivalent between the two groups (Table 5).

As shown in Table 5, the length of surgical time and amount of intraoperative blood loss were comparable between the LLR and OLR groups. There were fewer patients in the LLR group who required inflow control during the operation (42.5% vs. 65.8%, *p* = 0.005). Patients in the LLR group tended to have a shorter postoperative hospital stay than the OLR group (9.4 vs. 11.5 days, *p* = 0.071). Moreover, the LLR group had a lower postoperative complication rate than the OLR group (16.4% vs. 30.1%, *p* = 0.049). There was no mortality in the LLR group. As for tumor radicality, more than 50% of patients in both groups had their section margin larger than 0.5 cm. The oncological outcome in terms of DFS and OS, after PSM, was not different between the two groups (LLR vs. OLR: median DFS 69 ± 9.1 vs. 45 ± 5.3 months, *p* = 0.192; median OS 93 ± 2.9 vs. 86 ± 3.5 months, *p* = 0.146) (Figure 2C,D).

### 3.4. LLR and OLR for HCC Stratified by DS Groups

To further evaluate the feasibility and outcome of LLR in the various difficulty levels, the matched patients in the respective DS groups were compared and the results are shown in Table 6. The baseline clinical characteristics, tumor features, preoperative liver reserve, and resection extent were generally comparable across all DS groups, except that the LLR had a higher ICG-15 retention rate than the OLR in the advanced DS group (ICG-15 > 10%, 61.5% in the LLR and 15.4% in the OLR, *p* = 0.041), and the OLR had a larger tumor size than the LLR in the expert DS group (3.9 cm in the OLR group and 3.0 cm in the LLR group, *p* = 0.049). In the intermediate DS group, the LLR required less inflow control but longer duration of control as well as parenchymal transection (*p* = 0.076, 0.024, and 0.032, respectively). The other perioperative outcome including intraoperative blood loss, postoperative complications, 90-day mortality, and length of stay (LOS) were comparable between the LLR and OLR. Although not significant yet, there was a trend for a lower complication rate and shorter LOS in the LLR group.

**Figure 3:**
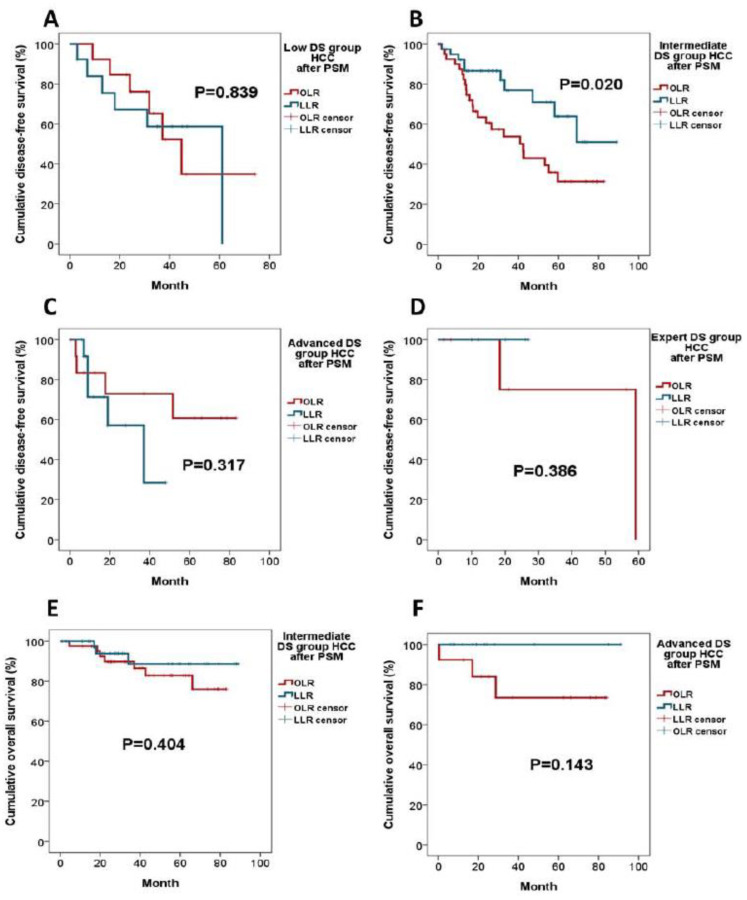
Kaplan–Meier survival analysis of LLR and OLR for HCC, stratified by IWATE difficulty score (DS) groups. (**A**) DFS of low DS group after PSM; (**B**) DFS of intermediate DS group after PSM; (**C**) DFS of advanced DS group after PSM; (**D**) DFS of expert DS group after PSM; (**E**) OS of intermediate DS group after PSM; and (**F**) OS of advanced DS group after PSM. All patients in the low and expert DS groups were still alive at the last follow-up.

The oncological survivals in respective DS groups after PSM are illustrated in Figure 3. The LLR enjoyed a significantly longer DFS than the OLR in the intermediate DS group (mean DFS 70.6 ± 6.8 vs. 58.9 ± 5.8 months, *p* = 0.020) (Figure 3B). The DFS in other DS groups were equivalent between the LLR and OLR. Likewise, the OS of the LLR and OLR were not significantly different; all patients in the low and expert DS groups were still alive at the last follow-up.

## 4. Discussion

Hepatocellular carcinoma is the most common primary malignancy of the liver and causes more than 8000 deaths annually in Taiwan [8,27,28,29]. Liver resection remains one of the most effective curative treatments for HCC and, with remarkable advancements in the field of laparoscopic surgery, the application of LLR for the treatment of HCC has also been discussed and initiated. To guide liver surgeons worldwide, consensus conferences were held and recommendations were proposed [1,15,16,17,30]. After the first international consensus conference for laparoscopic liver resection (ICCLLR), we reported our experience regarding laparoscopic left lateral sectionectomy (LLS) for HCC [7,30]. We demonstrated that LLS is safe for HCC, with surgical and oncological outcomes comparable to that of the conventional open approach. However, whether LLR is appropriate for HCC located at segments other than left lateral liver sector is still undetermined. A more objective investigation is mandatory to consolidate the role of LLR for HCC.

Compared to the conventional OLR, which usually adopts the Brisbane 2000 terminology of liver anatomy to classify the complexity of resection, the LLR is even more technically demanding and requires a more sophisticated classification or scoring system to categorize the difficulty of the liver resections [14]. Although major LLR was reported to have relatively worse outcome compared to minor resections in many studies, it may not be justified to compare the results of LLR and OLR based on this dichotomous classification [5]. The liver is a highly vascular solid organ, and, in many cases, HCC develops in the background of chronic liver diseases. Unlike other solid organ surgeries, the future liver remnant and liver functions are important factors that must be taken into consideration during liver resections. The IWATE criteria, which consist of vital variables including tumor size, location, extent of liver resection, liver function, proximity to major vessels, and the use of hybrid/hand-assisted laparoscopic procedure, were recently proposed to fulfill the urgent need for LLR. The validity of these criteria to assess the difficulty of LLR were subsequently confirmed by several recent studies [20,21,31]. Nevertheless, despite the affirmative results, few studies to date have adopted the IWATE criteria to compare the outcome between LLR and OLR for HCC, and none of them have employed PSM analysis to eliminate the influence of potential confounding factors. In the real world scenario, since liver surgeons might preferentially conduct LLR for less challenging HCC, the baseline characteristics are thus heterogeneous and the outcome analysis might be biased as a result. The current study, by adopting a 1:1 PSM analysis between the LLR and OLR, is one of the first studies in the English literature to report the surgical as well as the oncological outcome after surgery for HCC.

In the current study, we have demonstrated comparable surgical and oncological outcomes between the LLR and OLR with matched DS and baseline characteristics. The overall postoperative complications and LOS were all reduced in the LLR group. The extent of treatment radicality in terms of the width of the section margin was not compromised by the LLR, either. The subgroup analysis within individual DS groups also yielded similar results across all four groups. The index perioperative results including blood loss, surgical time, complications, LOS, section margin, and 90-day mortality were all equivalent between the LLR and OLR. Moreover, we have revealed, in addition to a comparable DFS in the other three DS groups, a significantly better DFS for LLR in the intermediate DS group. We believe this can be attributed to a magnified and clearer view under laparoscopy, which ensures a delicate control of the smaller vascular and biliary structures. The lesser extent of liver mobilization during LLR also has a contributing role. The potential spillage and/or spread of the microscopic tumor deposits over the remnant liver during parenchymal transection would thus be prevented. In short, we have shown that LLR should be a feasible and safe surgical approach for HCC and can provide a relatively consistent oncological outcome.

In addition to the current study, two recent landmark publications also demonstrated a better perioperative outcome and comparable long-term survival for LLR [32,33]. Despite similar results, there are several significant features that distinguish our research. First, the current study adopted the widely accepted and validated IWATE criteria to assess the patients. The categorization is more objective and reproducible. Second, not only the clinicopathological parameters but also the difficulties in terms of IWATE DS were matched between the two groups. We attempted to reduce the potential selection bias originating from different patient populations. Third, the results obtained from our study could be applied to the real world scenario; surgeons could comfortably allocate patients into a specific difficulty group and suggest appropriate treatment for them. Moreover, the types of liver resections were matched and there were fewer partial liver resections in the present cohort. Last but not the least, the operations analyzed in the current study were performed by the same surgical team, which rendered the surgical outcome more consistent. As a result, the current study should be an index research for liver surgeons worldwide.

Despite encouraging findings, we believe our results cannot be over extrapolated. As mentioned in the current study, a tumor located at S7/8 of liver was found to be independently associated with the development of postoperative complications. This corresponds to the IWATE criteria and other studies in which S7 and S8 were considered as the most challenging locations [34,35]. HCC situated herein would thus easily fall into either advanced or expert groups. Since postoperative complications were demonstrated to be predisposing factors for early HCC recurrence, efforts should be made to prevent the occurrence of complications [24]. As a result, similar to the recommendations concluded by the second ICCLLR, which stated that more complicated LLR was still in exploration phase and required more surgical skills, we believe a prospective randomized trial comparing LLR and OLR for HCC with higher DS should be undertaken to confirm our findings [15,16,17]. In the meantime, we believe we can only conclude that “laparoscopic liver resection should be a standard procedure for HCC with low or intermediate difficulty scores”.

In addition to tumor location, the current study also found preoperative anemia to be an independent risk factor for the occurrence of postoperative complications. This is supported by a previous report that preoperative anemia is independently associated with posthepatectomy morbidity [36]. Other clinical variables including systemic comorbidities, liver cirrhosis, and HCC, in contrast, were not significantly associated with the development of complications. As a result, HCC per se is not a relative contraindicator for LLR; on the contrary, LLR can provide a surgical and oncological outcome comparable to that of OLR. To prevent potential complications, however, LLR should be conducted with extreme caution in patients with anemia or difficult tumor location.

Aside from complications, HCC recurrence was also influenced by antiviral therapy in HCV-related HCC [37,38]. As a result, stringent surveillance and appropriate antiviral therapy are of paramount importance in this subset of patients. In the current study, 76.1% of HCV-HCC patients received antiviral therapy (77.1% interferon-based, and 22.9% direct antiviral agent-based therapy), and the administration of antiviral therapy was not associated with the development of tumor recurrence (48.6% in the treated group vs. 27.3% in the untreated group, *p* = 0.188). However, there was still a trend toward recurrence in the treated group. Further studies are thus warranted to determine the influence of antiviral therapy on the recurrence of HCV-related HCC.

As mentioned above, liver cirrhosis was not significantly associated with the occurrence of complications in LLR. In addition, there are studies showing that, in patients with cirrhosis, LLR might be even better than OLR in terms of the perioperative results [39]. Recent research by Troisi et al. demonstrated that LLR can be safely performed in selected Child B cirrhosis patients with fewer postoperative complications and ascites [32]. As a result, we believe in certain Child B patients, such as those with mild impairment of liver function, without preoperative portal hypertension, or with Child B7 cirrhosis, LLR should be considered when only limited resection is planned. Further prospective trials are necessary to clarify the role of LLR in patients with Child B cirrhosis.

The current study still has several limitations. First, the sample size of LLR was limited, especially those in the advanced or expert DS groups. Second, the follow-up duration was not long enough, which rendered the survival analysis less significant [40]. Third, the current study lacked sufficient data regarding the suitability of LLR for large (>5 cm) or huge (>10 cm) HCC. The benefit of LLR for larger HCC thus cannot be concluded from our results. Moreover, in the subgroup analysis, the LLR had a higher ICG-15 retention rate than the OLR in the advanced DS group (ICG-15 > 10%, 61.5% in the LLR and 15.4% in the OLR, *p* = 0.041) and the OLR had a larger tumor size than the LLR in the expert DS group (3.9 cm in the OLR group and 3.0 cm in the LLR group, *p* = 0.049). These confounding factors may bias the analysis and interfere with the final survival outcome. Last but not least, although PSM analysis is generally acknowledged for its capability of reducing selection bias in retrospective studies, the estimation could still be deviated if there were any neglected confounding factors. In the current study, for example, the influence of the hepatitis viral load was not considered since many patients did not have their viral load checked in the first place. Therefore, further well-designed and larger scale prospective randomized trials comparing LLR and OLR for HCC with longer follow-up are mandatory to consolidate the role of LLR for HCC.

## 5. Conclusions

Our study demonstrated the surgical and oncological outcome of HCC following LLR. With sufficient experience and adequate preoperative preparation, LLR for HCC is feasible and the perioperative outcome is favorable. Based on the current study, we suggest LLR should be a standard procedure for HCC with low or intermediate IWATE difficulty scores. It can provide satisfactory postoperative recovery and comparable oncological outcomes. Due to limited sample size and potential confounding factors, further larger scale prospective studies are warranted to validate our findings.

## Figures and Tables

**Table 1:** Demographic features of patients (*n* = 138) undergoing laparoscopic liver resections ^a^.

Variables	*n* (%)	Variables	*n* (%)
Male gender	90 (65.2)	Tumor size > 3 cm	69 (50.0)
Age ≥ 65 years	46 (33.3)	Tumor size > 5 cm	22 (15.9)
Diagnosis		Tumor location at S7/8	28 (20.3)
Hepatocellular carcinoma	81 (58.7)	Proximity to major vessels	40 (29.0)
Intrahepatic cholangiocarcinoma	4 (2.9)	Difficulty score group	
Benign hepatic tumor ^b^	32 (15.2)	Low (score 0–3)	26 (18.8)
Biliary/hepatic cystic lesion	10 (7.2)	Intermediate (score 4–6)	72 (52.2)
Metastatic liver tumor	15 (10.9)	Advanced (score 7–9)	29 (21.0)
Other indication	7 (5.1)	Expert (score 10–13)	11 (8.0)
Procedure type		Postoperative complications ^f^	
Right hepatectomy	10 (7.2)	Grade I and II	17 (12.3)
Left hepatectomy	6 (4.3)	≥Grade III	3 (2.2)
Left lateral sectionectomy	37 (26.8)	Follow up status	
Tri-segmentectomy ^c^	6 (4.3)	Alive	121 (87.7)
Bi-segmentectomy ^d^	9 (6.5)	Expired	9 (6.5)
Segmentectomy	30 (21.7)	Loss of follow up	8 (5.8)
Partial hepatectomy	40 (29.0)	Age (year) (mean ± SD ^g^ (range))	57.7 ± 12.95 (30–84)
Major resection ^e^	22 (15.9)	Tumor size (mean ± SD ^g^ (range))	3.78 ± 2.65 (0.9–17.0)

^a^ performed by a single surgeon; ^b^ includes hepatic adenoma, focal nodular hyperplasia, hemangioma; ^c^ includes central lobectomy; ^d^ excludes left lateral sectionectomy ^e^ resection of more than 3 segments; ^f^ Clavien–Dindo classifications. ^g^ standard deviation.

**Table 2:** Risk factors for postoperative complications after laparoscopic liver resections.

Variables	Univariate			Multivariate	
	Complications (%(*n*))	Odds Ratio	*p* Value	Hazard Ratio (95%CI ^a^)	*p* Value
Age (≤65 vs. >65)	21.7 (10) vs. 12.0 (11)	1.81	0.131	-	-
Sex (male vs. female)	15.6 (14) vs. 14.9 (7)	1.04	0.919	-	-
DM ^b^ (yes vs. no)	30.8 (8) vs. 11.7 (13)	2.62	0.015	2.03 (0.64–6.45)	0.230
Hypertension (yes vs. no)	26.8 (11) vs. 10.4 (10)	2.58	0.015	2.47 (0.81–7.52)	0.111
ESRD ^c^ (yes vs. no)	0.0 (0) vs. 15.4 (21)	N.A.	0.669	-	-
Cirrhosis (yes vs. no)	25.0 (10) vs. 11.3 (11)	2.20	0.044	2.20 (0.77–6.33)	0.143
Smoking (yes vs. no)	17.4 (4) vs. 14.9 (17)	1.17	0.768	-	-
Alcohol (yes vs. no)	18.2 (4) vs. 14.8 (17)	1.23	0.685	-	-
Previous abdominal surgery (yes vs. no)	24.0 (6) vs. 13.6 (15)	1.76	0.197	-	-
HCC ^d^ (yes vs. no)	16.0 (13) vs. 14.0 (8)	1.14	0.813	-	-
Hemoglobin (≤12 vs. >12 (g/dL))	33.3 (9) vs. 10.9 (12)	3.06	0.004	4.29 (1.37–13.33)	0.012
Platelet (≤150 vs. >150 (1000/uL))	20.0 (7) vs. 13.7 (14)	1.46	0.374	-	-
Bilirubin total (>1.2 vs. ≤1.2 (mg/dL))	22.2 (2) vs. 14.8 (19)	1.50	0.553	-	-
Albumin (≤3.5 vs. >3.5(g/dL))	30.0 (3) vs. 13.8 (17)	2.17	0.174	-	-
α-fetoprotein (>200 vs. ≤200 (ng/mL))	14.3 (3) vs. 14.4 (13)	0.99	0.985	-	-
ICG-15 clearance (>10 vs. ≤10 (%))	21.6 (8) vs. 10.3 (7)	2.10	0.113	-	-
Tumor size (>5 vs. ≤5 (cm))	9.1 (2) vs. 16.7 (19)	0.54	0.526	-	-
Tumor size (>3 vs. ≤3 (cm))	11.8 (8) vs. 18.8 (13)	0.62	0.250	-	-
Tumor location (seg 7/8 vs. other segs)	28.6 (8) vs. 11.9 (13)	2.40	0.029	4.26 (1.32–13.70)	0.015
Extent of liver resection (not less than sectionectomy vs. others)	22.6 (7) vs. 13.2 (14)	1.71	0.203	-	-
Proximity to major vessels (yes vs. no)	17.9 (7) vs. 15.6 (14)	1.15	0.735	-	-
Resection type (major vs. minor) ^e^	23.8 (5) vs. 13.8 (16)	1.73	0.241	-	-
Inflow control (yes vs. no)	22.6 (12) vs. 10.7 (9)	2.11	0.059	-	-
Operation duration (>200 vs. ≤200 (min))	15.6 (17) vs. 14.8 (4)	1.05	1.000	-	-
Blood loss (>500 vs. ≤500 (mL))	21.7 (5) vs. 14.2 (16)	1.53	0.359	-	-
Difficulty score group					
Intermediate vs. Low	16.7 (12) vs. 7.7 (2)	2.16	0.342	-	-
Advanced vs. Low	13.8 (4) vs. 7.7 (2)	1.79	0.672	-	-
Expert vs. Low	27.3 (3) vs. 7.7 (2)	3.54	0.144	-	-

^a^ confidence interval; ^b^ diabetes mellitus; ^c^ end-stage renal disease; ^d^ hepatocellular carcinoma; ^e^ major resection: resection of more than 3 segments.

**Table 3:** Clinical characteristics of hepatocellular carcinoma patients who received either LLR ^a^ or OLR ^b^.

Variables	LLR (*n* = 81(100%))	OLR (*n* = 526(100%))	*p* Value
Age (year) (mean ± SD)	60.2 ± 12.2	61.9 ± 11.2	0.162
Male gender (*n* (%))	61 (75.3)	412 (78.3)	0.542
Previous abdominal surgery (*n* (%))	9 (11.1)	145 (27.6)	0.001
DM ^c^ (*n* (%))	17 (21.0)	142 (27.0)	0.252
Hypertension (*n* (%))	25 (30.9)	221 (42.0)	0.057
ESRD ^d^ (*n* (%))	0 (0.0)	12 (2.3)	0.384
HBV infection (*n* (%))	47 (58.0)	313 (60.0)	0.870
HCV infection (*n* (%))	29 (35.8)	135 (26.0)	0.173
Cirrhosis (*n* (%))	36 (44.4)	254 (48.5)	0.499
Hemoglobin (g/dL) (mean ± SD)	13.5 ± 1.8	13.5 ± 1.9	0.832
Albumin (g/dL) (mean ± SD)	4.17 ± 0.41	4.15 ± 0.42	0.610
ICG-15 > 10% (*n* (%))	32 (39.5)	211 (41.4)	0.751
α-fetoprotein > 200 ng/mL (*n* (%))	21 (27.6)	123 (23.4)	0.417
Child–Pugh classification (*n* (%))			1.000
A	81 (100.0)	519 (99.4)	
B	0 (0.0)	3 (0.6)	
Tumor size (cm) (mean ± SD)	3.16 ± 1.44	5.13 ± 3.83	<0.001
Tumor size > 3 cm (*n* (%))	40 (49.4)	342 (65.0)	0.007
Tumor size > 5 cm (*n* (%))	6 (7.4)	182 (34.6)	<0.001
Tumor location at seg 7/8 (*n* (%))	14 (17.3)	267 (50.8)	<0.001
Major resection ^e^ (*n* (%))	12 (14.8)	184 (35.0)	<0.001
Difficulty score group (*n* (%))			<0.001
Low (score 0–3)	16 (19.8)	21 (4.0)	
Intermediate (score 4–6)	44 (54.3)	136 (25.9)	
Advanced (score 7–9)	15 (18.5)	211 (40.1)	
Expert (score 10–13)	6 (7.4)	158 (30.0)	
Postoperative complications ^f^ (any grade, *n* (%))	13 (16.0)	185 (35.2)	0.001
Grade ≧III complications ^f^ (*n* (%))	2 (2.5)	49 (9.3)	0.049
90-day mortality (*n* (%))	0 (0.0)	8 (1.5)	0.606
Surgical time (min) (mean ± SD)	289.7 ± 99.8	312.9 ± 109.2	0.072
Blood loss (mL) (mean ± SD)	336.3 ± 379.0	575.0 ± 911.1	<0.001
Postoperative length of hospital stay (day) (mean ± SD)	9.3 ± 5.1	12.0 ± 8.7	<0.001
Vascular invasion (*n* (%))	20 (24.7)	211 (40.2)	0.008
Daughter nodules (*n* (%))	25 (30.9)	75 (14.3)	<0.001
Encapsulation (present, *n* (%))	75 (92.6)	449 (85.4)	0.078
Capsule invasion (*n* (%))	51 (63%)	342 (65%)	0.718
Section margin > 0.5 cm (*n* (%))	47 (58.0)	219 (43.2)	0.013
Positive resection margin (*n* (%))	1 (1.2)	20 (3.8)	0.339
AJCC T Stage (*n* (%))			0.006
T1	51 (62.5)	250 (47.6)	
T2	26 (32.5)	159 (30.2)	
T3a	0 (0.0)	32 (6.1)	
T3b	2 (2.5)	35 (6.7)	
T4	2 (2.5)	50 (9.5)	

^a^ laparoscopic liver resection; ^b^ open liver resection; ^c^ diabetes mellitus; ^d^ end-stage renal disease; ^e^ resection of more than 3 segments; ^f^ Clavien–Dindo classifications.

**Table 4:** Clinical characteristics of hepatocellular carcinoma patients after propensity-score matching between the LLR ^a^ and OLR ^b^.

	LLR (*n* = 73(100%))	OLR (*n* = 73(100%))	*p* Value
Age (yr) (mean ± SD)	60.4 ± 11.7	58.2 ± 11.1	0.237
Male gender (*n (*%))	54 (74.0)	60 (82.2)	0.230
DM ^c^ (*n (*%))	14 (19.2)	14 (19.2)	1.000
Hypertension (*n (*%))	22 (30.1)	22 (30.1)	1.000
ESRD ^d^ (*n (*%))	0 (0.0)	0 (0.0)	1.000
HBV infection (*n (*%))	51 (69.9)	41 (56.2)	0.086
HCV infection (*n (*%))	29 (38.4)	17 (23.6)	0.090
Cirrhosis (*n (*%))	33 (45.2)	36 (49.3)	0.619
Child–Pugh class A (*n (*%))	73 (100.0)	73 (100.0)	1.000
Hemoglobin < 12 g/dL (*n (*%))	14 (19.2)	12 (16.4)	0.665
Albumin (g/dL) (mean ± SD)	4.16 ± 0.41	4.19 ± 0.39	0.680
ICG-15 > 10% (*n (*%))	32 (43.8)	26 (36.6)	0.377
α-fetoprotein > 200 ng/mL (*n (*%))	18 (26.5)	15 (20.5)	0.407
Tumor size (cm) (mean ± SD)	3.24 ± 1.46	3.23 ± 1.57	0.952
Tumor size > 3 cm (*n (*%))	38 (52.1)	32 (43.2)	0.320
Tumor size > 5 cm (*n (*%))	6 (8.2)	8 (11.0)	0.574
Tumor location at seg 7/8 (*n (*%))	14 (19.2)	14 (19.2)	1.000
Proximity to major vessels <1 cm (*n (*%))	17 (23.3)	18 (24.7)	0.846
Previous abdominal surgery (*n (*%))	7 (9.6)	9 (12.3)	0.596
Major resection ^e^ (*n (*%))	10 (13.7)	9 (12.3)	0.806
Difficulty score group (*n (*%))			1.000
Low (score 0–3)	13 (17.8)	13 (17.8)	
Intermediate (score 4–6)	41 (56.2)	41 (56.2)	
Advanced (score 7–9)	13 (17.8)	13 (17.8)	
Expert (score 10–13)	6 (8.2)	6 (8.2)	

^a^ laparoscopic liver resection ^b^ open liver resection ^c^ diabetes mellitus ^d^ end-stage renal disease. ^e^ resection of more than 3 segments.

**Table 5:** Comparison of perioperative and oncologic outcomes after propensity-score matching between the LLR ^a^ and OLR ^b^.

	LLR (*n* = 73(100%))	OLR (*n* = 73(100%))	*p* Value
Inflow control (yes, (*n (*%))	31 (42.5)	48 (65.8)	0.005
Duration of inflow control (min) (mean ± SD)	70.4 ± 36.2	51.4 ± 23.4	0.022
Postoperative complications ^c^ (any grade, *n (*%))	12 (16.4)	22 (30.1)	0.049
Grade III complication ^c^ (*n (*%))	2 (2.7)	7 (9.6)	0.166
90-day mortality (*n (*%))	0 (0.0)	2 (2.7)	0.497
Surgical time (min) (mean ± SD)	288.6 ± 102.1	276.0 ± 106.7	0.501
Surgical time > 300 min	28 (38.4)	26 (35.6)	0.732
Blood loss (mL) (mean ± SD)	342.5 ± 394.4	400.7 ± 531.9	0.456
Blood loss > 500 mL (*n (*%))	13 (18.1)	17 (23.3)	0.437
Postoperative length of stay (LOS) (day) (mean ± SD)	9.4 ± 5.3	11.5 ± 9.8	0.071
LOS ≤ 7 days (*n (*%))	29 (39.7)	22 (30.1)	0.224
Encapsulation (present, *n (*%))	67 (91.8)	59 (80.8)	0.054
Capsular invasion (*n (*%))	44 (60.3)	44 (60.3)	1.000
Tumor rupture (*n (*%))	3 (4.1)	3 (4.1)	1.000
Vascular invasion (*n (*%))	19 (26.1)	21 (28.8)	0.711
Daughter nodules (*n (*%))	25 (34.2)	4 (5.5)	<0.001
Section margin (*n (*%))			0.454
Positive margin	1 (1.4)	4 (5.5)	
<0.5 cm	31 (42.5)	28 (38.4)	
0.5–0.9 cm	14 (19.2)	20 (27.4)	
1–1.9 cm	17 (23.3)	13 (17.8)	
≧2 cm	10 (13.7)	8 (11.0)	
Section margin > 0.5 cm (*n (*%))	41 (56.2)	41 (56.2)	1.000
Positive resection margin (*n (*%))	1 (1.4)	4 (5.5)	0.172
Edmondson-Steiner grade (III and IV, *n (*%))	31 (43.1)	25 (35.2)	0.393
AJCC T Stage (*n (*%))			0.519
T1	46 (63.0)	46 (63.0)	
T2	23 (31.5)	23 (31.5)	
T3a	0 (0.0)	0 (0.0)	
T3b	2 (2.7)	1 (1.4)	
T4	2 (2.7)	3 (4.1)	
Follow up status			0.179
Alive	64 (87.7)	58 (79.5)	
Die from the disease	3 (4.1)	10 (13.7)	
Die from other disease	1 (1.4)	2 (2.7)	
Loss of follow up	5 (6.8)	3 (4.1)	

^a^ laparoscopic liver resection ^b^ open liver resection ^c^ Clavien–Dindo classifications.

**Table 6:** Comparison of perioperative and oncologic outcomes after propensity-score matching between the LLR ^a^ and OLR ^b^, stratified by the IWATE difficulty groups.

Difficulty Group	Low (*n* = 13)			Intermediate (*n* = 41)			Advanced (*n* = 13)			Expert (*n* = 6)		
	LLR	OLR	*p*-Value	LLR	OLR	*p*-Value	LLR	OLR	*p*-Value	LLR	OLR	*p*-Value
Age (mean ± SD ^c^)	64.0 ± 11.4	61.7 ± 8.9	0.572	60.4 ± 12.3	56.6 ± 11.5	0.158	60.7 ± 9.8	59.3 ± 12.9	0.765	53.2 ± 10.2	59.5 ± 8.4	0.272
Sex (Male (*n (*%))	8 (61.5)	10 (76.9)	0.673	33 (80.5)	34 (82.9)	1.000	10 (76.9)	11 (84.6)	1.000	3 (50.0)	5 (83.3)	0.545
DM ^d^ (Yes (*n (*%))	3 (23.1)	3 (23.1)	1.000	8 (19.5)	8 (19.5)	1.000	2 (15.4)	2 (15.4)	1.000	1 (16.7)	1 (16.7)	1.000
Hypertension (Yes (*n (*%))	5 (38.5)	6 (46.2)	0.691	9 (22.0)	9 (22.0)	1.000	6 (46.2)	5 (38.5)	0.691	2 (33.3)	2 (33.3)	0.691
HBV infection (*n (*%))	7 (53.8)	7 (53.8)	1.000	24 (58.5)	30 (73.2)	0.244	7 (53.8)	10 (76.9)	0.411	3 (50.0)	4 (66.7)	1.000
HCV infection (*n (*%))	7 (53.8)	3 (25.0)	0.226	13 (31.7)	11 (26.8)	0.632	5 (38.5)	2 (15.4)	0.378	3 (50.0)	1 (16.7)	0.545
Cirrhosis (Yes (*n (*%))	7 (53.8)	6 (46.2)	0.695	17 (41.5)	21 (51.2)	0.507	6 (46.2)	6 (46.2)	1.000	3 (50.0)	3 (50.0)	1.000
Tumor size (mean ± SD ^c^)	2.3 ± 0.8	2.2 ± 0.4	0.734	3.4 ± 1.7	3.1 ± 1.6	0.339	3.7 ± 1.2	4.4 ± 1.6	0.241	3.0 ± 0.7	3.9 ± 0.7	0.049
Tumor size > 5 cm (*n (*%))	0 (0.0)	0 (0.0)	1.000	4 (9.8)	4 (9.8)	1.000	2 (15.4)	4 (30.8)	0.645	0 (0.0)	0 (0.0)	1.000
Tumor location at S7/8 (*n (*%))	0 (0.0)	0 (0.0)	1.000	6 (14.6)	6 (14.6)	1.000	2 (15.4)	2 (15.4)	1.000	6 (100.0)	6 (100.0)	1.000
Proximity to major vessels < 1 cm (*n (*%))	1 (7.7)	1(7.7)	1.000	5 (12.2)	6(14.6)	0.746	7 (53.8)	6 (46.2)	0.695	4 (66.7)	5 (83.8)	1.000
Major resection ^e^ (*n (*%))	0 (0.0)	0 (0.0)	1.000	1 (2.4)	0 (0.0)	1.000	8 (61.5)	8 (61.5)	1.000	4 (66.7)	4 (66.7)	1.000
Hb ^f^ < 12 (g/dL)	2 (15.4)	1 (7.7)	1.000	9 (22.0)	9 (22.0)	1.000	2 (15.4)	2 (15.4)	1.000	1 (16.7)	0 (0.0)	1.000
ICG-15 > 10% (%)	6 (46.2)	8 (61.5)	0.431	16 (39.0)	16 (39.0)	1.000	8 (61.5)	2 (15.4)	0.041	2 (33.3)	2 (33.3)	1.000
Albumin (g/dL) (mean ± SD ^c^)	4.30 ± 0.33	4.31 ± 0.28	0.959	4.11 ± 0.46	4.16 ± 0.43	0.597	4.15 ± 0.32	4.09 ± 0.38	0.634	4.28 ± 0.35	4.39 ± 0.26	0.572
Bilirubin (mg/dL) (mean ± SD ^c^)	0.65 ± 0.23	0.72 ± 0.33	0.541	0.71 ± 0.30	0.67 ± 0.31	0.573	0.86 ± 0.51	0.76 ± 0.20	0.610	0.65 ± 0.28	0.76 ± 0.30	0.513
Inflow control (yes (*n (*%))	3 (23.1)	8 (61.5)	0.111	15 (36.6)	23 (56.1)	0.076	10 (76.9)	12 (92.3)	0.593	3 (50.0)	5 (83.3)	0.545
Duration of control (min) (mean ± SD ^c^)	59.0 ± 28.6	41.1 ± 22.9	0.293	63.8 ± 28.7	46.0 ± 17.3	0.024	84.1 ± 44.3	62.8 ± 28.1	0.200	69.0 ± 65.1	75.0 ± 21.2	0.862
Postoperative complications ^g^ (any grade, *n (*%))	1 (7.7)	4 (30.8)	0.322	6 (14.6)	9 (22.0)	0.391	2 (15.4)	5 (38.5)	0.378	3 (50.0)	4 (66.7)	0.558
Grade ≧III complication ^f^ (*n (*%))	0 (0.0)	1 (7.7)	1.000	0 (0.0)	1 (2.4)	1.000	1 (7.7)	3 (23.1)	0.593	1 (16.7)	2 (33.3)	1.000
90-day mortality (*n (*%))	0 (0.0)	0 (0.0)	1.000	0 (0.0)	0 (0.0)	1.000	0 (0.0)	1 (7.7)	1.000	0 (0.0)	1 (16.7)	1.000
Surgical time (min) (mean ± SD ^c^)	211.7 ± 67.8	249.0 ± 106.3	0.298	278.1 ± 92.6	246.7 ± 76.9	0.133	352.4 ± 87.0	339.9 ± 126.1	0.770	388.5 ± 115.1	407.5 ± 106.0	0.772
Transection time (min) (mean ± SD ^c^)	69.0 ± 42.6	54.8 ± 31.9	0.360	87.2 ± 46.1	66.8 ± 31.0	0.032	120.0 ± 39.7	93.4 ± 32.0	0.073	155.0 ± 83.8	136.6 ± 39.3	0.638
Blood loss (mL) (mean ± SD ^c^)	242.3 ± 228.5	234.6 ± 234.8	0.933	274.7 ± 272.8	321.9 ± 279.3	0.444	620.0 ± 650.6	465.3 ± 319.1	0.449	410.0 ± 467.8	550.0 ± 200.0	0.551
Blood loss > 500 mL (*n (*%))	1 (7.7)	1 (7.7)	1.000	5 (12.5)	7 (17.1)	0.562	5 (38.5)	5 (38.5)	1.000	2 (33.3)	4 (66.7)	0.567
Postoperative length of stay (LOS) (day) (mean ± SD ^c^)	7.7 ± 2.5	9.2 ± 3.3	0.186	8.9 ± 5.0	9.6 ± 4.8	0.111	11.5 ± 6.3	10.8 ± 4.7	0.840	12.5 ± 8.3	14.4 ± 7.0	0.394
LOS ≦ 7 days (*n (*%))	7 (53.8)	6(46.2)	0.695	19 (46.3)	11 (26.8)	0.067	2 (15.4)	4 (30.8)	0.645	1 (16.7)	1 (16.7)	1.000
Section margin > 0.5 cm (*n (*%))	8 (61.5)	8 (61.5)	1.000	22 (53.7)	25 (61.0)	0.503	9 (69.2)	4 (30.4)	0.115	2 (33.3)	4 (66.7)	0.567
Positive resection margin (*n (*%))	0 (0.0)	1 (7.7)	1.000	1 (7.7)	2 (4.9)	1.000	0 (0.0)	0 (0.0)	1.000	0 (0.0)	1 (16.7)	1.000

^a^ laparoscopic liver resection ^b^ open liver resection ^c^ standard deviation ^d^ diabetes mellitus ^e^ resection of more than 3 segments. ^f^ hemoglobin ^g^ Clavien–Dindo classifications.

## Data Availability

All data generated or analyzed during the study are included in this published article. Raw data may be requested from the authors with the permission of the institution.

## Abbreviations

AJCC	American Joint Committee on Cancer
CGMH	Chang Gung memorial hospital
CI	confidence interval
DFS	disease-free survival
DM	diabetes mellitus
DS	difficulty score
ESRD	end-stage renal disease
Hb	hemoglobin
HBV	hepatitis B virus
HCV	hepatitis C virus
HCC	hepatocellular carcinoma
HALS	hand-assisted laparoscopic surgery
ICCLLR	international consensus conference on LLR
ICG-15	indocyanine green retention test at 15 min
IRB	Institutional Review Board
LLR	laparoscopic liver resection
LOS	length of stay
OLR	open liver resection
OS	overall survival
PSM	propensity-score matched
SD	standard deviation
